# Human Factors Linked with Initial and Continuous Trust in Autonomous Systems: A Literature Review

**DOI:** 10.1093/geroni/igab046.2485

**Published:** 2021-12-17

**Authors:** Maria Pena, Jared Carrillo, Nonna Milyavskaya, Thomas Chan

**Affiliations:** 1 California State University Northridge, Pacoima, California, United States; 2 California State University Northridge, Van Nuys, California, United States; 3 California State University Northridge, Northridge, California, United States

## Abstract

Many autonomous systems are being developed to assist older adults to age in place. However, there is little research related to the human factors associated with why older adults may initially and continuously trust these autonomous systems. More research in this area on older adults and trust in autonomy is needed to facilitate the technologies better everyday use. The current study conducted a literature review on the prevalent human factors that enable people to trust their interactions with smart technologies (e.g., artificial intelligence, navigational structures). Articles were collected from various disciplines on concepts such as trust in autonomy, human-computer interactions and teamwork. Thematic analysis revealed two convergent areas that were associated with initial and continuous trust: human and technological characteristics. Human characteristics are defined by a person’s ability to understand and use autonomous systems. Generally, people with higher competency and abilities with autonomous systems demonstrated the ease of use to carry out desired actions with smart technology. Technological characteristics are defined by the system’s performance, explainability, and its intended purpose between trust. Essentially, people were less critical of autonomous systems that were perceived to be useful, transparent, and predictable. Overall, the autonomous system's ability to perform its intended purpose and the users knowledge and technical qualifications dominate the relationship between initial and continuous trust with autonomous systems. These are the prevalent factors that need to be considered for the creation of trusted autonomous technologies for older adults to help them age in the approaching more advanced technological world.

